# Association between early cumulative fluid balance and successful liberation from invasive ventilation in COVID-19 ARDS patients — insights from the PRoVENT-COVID study: a national, multicenter, observational cohort analysis

**DOI:** 10.1186/s13054-022-04023-y

**Published:** 2022-06-01

**Authors:** Sanchit Ahuja, Harm-Jan de Grooth, Frederique Paulus, Fleur L. van der Ven, Ary Serpa Neto, Marcus J. Schultz, Pieter R. Tuinman, PRoVENT-COVID Study Collaborative Group* ‘PRactice of VENTilation in COVID–19’, S. Ahuja, J. P. van Akkeren, A. G. Algera, C. K. Algoe, R. B. van Amstel, A. Artigas, O. L. Baur, P. van de Berg, A. E. van den Berg, D. C. J. J. Bergmans, D. I. van den Bersselaar, F. A. Bertens, A. J. G. H. Bindels, M. M. de Boer, S. den Boer, L. S. Boers, M. Bogerd, L. D. J. Bos, M. Botta, J. S. Breel, H. de Bruin, S. de Bruin, C. L. Bruna, L. A. Buiteman-Kruizinga, O. L. Cremer, R. M. Determann, W. Dieperink, D. A. Dongelmans, H. S. Franke, M. S. Galek-Aldridge, M. J. de Graaff, L. A. Hagens, J. J. Haringman, S. T. van der Heide, P. L. J. van der Heiden, N. F. L. Heijnen, S. J. P. Hiel, L. L. Hoeijmakers, L. Hol, M. W. Hollmann, M. E. Hoogendoorn, J. Horn, R. van der Horst, E. L. K. Ie, D. Ivanov, N. P. Juffermans, E. Kho, E. S. de Klerk, A. W. M. M. Koopman-van Gemert, M. Koopmans, S. Kucukcelebi, M. A. Kuiper, D. W. de Lange, N. van Mourik, S. G. Nijbroek, M. Onrust, E. A. N. Oostdijk, F. Paulus, C. J. Pennartz, J. Pillay, L. Pisani, I. M. Purmer, T. C. D. Rettig, J. P. Roozeman, M. T. U. Schuijt, M. J. Schultz, A. Serpa Neto, M. E. Sleeswijk, M. R. Smit, P. E. Spronk, W. Stilma, A. C. Strang, A. M. Tsonas, P. R. Tuinman, C. M. A. Valk, F. L. Veen-Schra, L. I. Veldhuis, P. van Velzen, W. H. van der Ven, A. P. J. Vlaar, P. van Vliet, P. H. J. van der Voort, L. van Welie, H. J. F. T. Wesselink, H. H. van der Wier-Lubbers, B. van Wijk, T. Winters, W. Y. Wong, A. R. H. van Zanten

**Affiliations:** 1 Department of Anesthesiology, Pain Management and Perioperative Medicine, Henry Ford Hospital, Detroit, MI USA; 2 Outcomes Research Consortium, Cleveland Clinic, Cleveland, OH USA; 3 Department of Intensive Care, Amsterdam UMC, Location VU Medical Center, Amsterdam, The Netherlands; 4 Department of Intensive Care, C3–415, Amsterdam UMC, Location AMC, Meibergdreef 9, 1105 AZ Amsterdam, The Netherlands; 5 ACHIEVE, Faculty of Health, Centre of Applied Research, Amsterdam University of Applied Sciences, Amsterdam, The Netherlands; 6 Department of Critical Care Medicine, Melbourne Medical School, University of Melbourne, Austin Hospital, Melbourne, Australia; 7 Australian and New Zealand Intensive Care Research Centre (ANZIC-RC), School of Public Health and Preventive Medicine, Monash University, Melbourne, Australia; 8 Department of Critical Care Medicine, Hospital Israelita Albert Einstein, São Paulo, Brazil; 9 Mahidol Oxford Tropical Medicine Research Unit (MORU), Mahidol University, Bangkok, Thailand; 10 Nuffield Department of Medicine, University of Oxford, Oxford, UK

**Keywords:** Cumulative fluid balance, Liberation of ventilation, COVID-19, ARDS, Critical care

## Abstract

**Background:**

Increasing evidence indicates the potential benefits of restricted fluid management in critically ill patients. Evidence lacks on the optimal fluid management strategy for invasively ventilated COVID-19 patients. We hypothesized that the cumulative fluid balance would affect the successful liberation of invasive ventilation in COVID-19 patients with acute respiratory distress syndrome (ARDS).

**Methods:**

We analyzed data from the multicenter observational ‘PRactice of VENTilation in COVID-19 patients’ study. Patients with confirmed COVID-19 and ARDS who required invasive ventilation during the first 3 months of the international outbreak (March 1, 2020, to June 2020) across 22 hospitals in the Netherlands were included. The primary outcome was successful liberation of invasive ventilation, modeled as a function of day 3 cumulative fluid balance using Cox proportional hazards models, using the crude and the adjusted association. Sensitivity analyses without missing data and modeling ARDS severity were performed.

**Results:**

Among 650 patients, three groups were identified. Patients in the higher, intermediate, and lower groups had a median cumulative fluid balance of 1.98 L (1.27–7.72 L), 0.78 L (0.26–1.27 L), and − 0.35 L (− 6.52–0.26 L), respectively. Higher day 3 cumulative fluid balance was significantly associated with a lower probability of successful ventilation liberation (adjusted hazard ratio 0.86, 95% CI 0.77–0.95, *P* = 0.0047). Sensitivity analyses showed similar results.

**Conclusions:**

In a cohort of invasively ventilated patients with COVID-19 and ARDS, a higher cumulative fluid balance was associated with a longer ventilation duration, indicating that restricted fluid management in these patients may be beneficial.

*Trial registration* Clinicaltrials.gov (NCT04346342); Date of registration: April 15, 2020.

**Graphical abstract:**

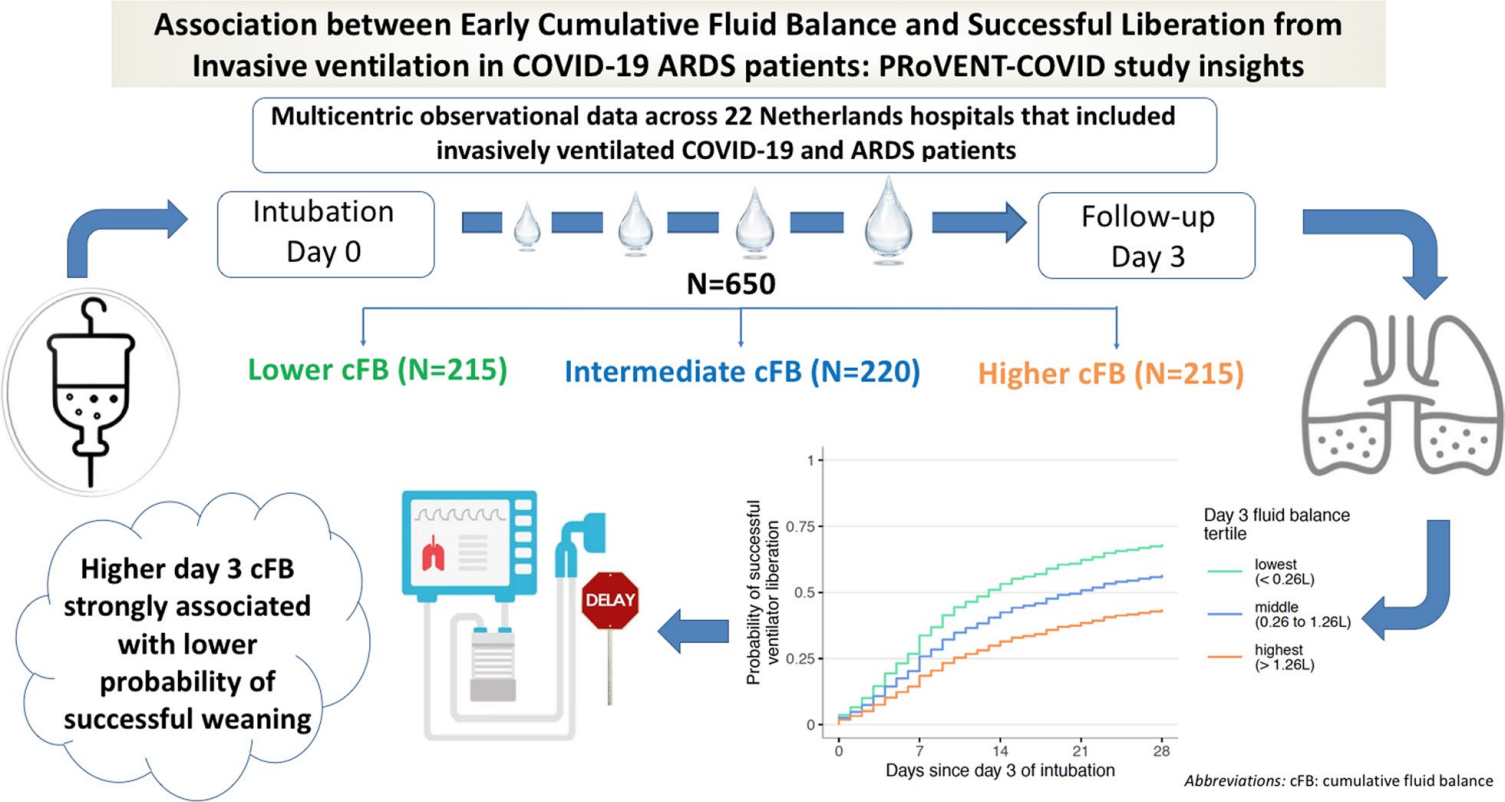

**Supplementary Information:**

The online version contains supplementary material available at 10.1186/s13054-022-04023-y.

## Background

Acute respiratory failure necessitating invasive ventilation is considered one of the leading causes of death in patients with COVID-19 [1]. Intravenous fluid therapy remains one of the cornerstones of resuscitation for nearly all forms of shock. While early fluid resuscitation is critical in managing shock, the accumulation of positive fluid balance has also been associated with worsening acute respiratory distress syndrome (ARDS). A previous study of non-COVID-19 patients with ARDS suggests that a higher positive cumulative fluid balance is independently associated with mortality, longer ventilation duration, and extended intensive care unit (ICU) stay [2]. Based on this indirect evidence, consensus guidelines during the early stages of the pandemic on the management of shock in patients with COVID-19 recommended targeting a neutral fluid balance strategy [3].

Considerable evidence from observational studies, clinical trials, and systematic reviews indicates the potential benefits of restricting fluid administration in critically ill patients [4–7]. Excessive fluid administration may increase the risk of pulmonary complications and the effects of edema in vital organs, causing injury [8, 9]. On the other hand, a restrictive fluid strategy could lead to extrapulmonary organ dysfunction consequent to reduced cardiac output; however, more recent evidence suggests mixed results in critically ill patients [10, 11]. Therefore, the fluid balance being an adverse prognostic factor, yet also a potentially modifiable risk factor, poses a unique dilemma in the management of critically ill COVID-19 patients. Current evidence is insufficient and constantly evolving to best address the optimal fluid management strategy in invasively ventilated COVID-19 patients. Using the database of the multicenter observational ‘PRactice of VENTilation in COVID-19 patients’ (PRoVENT-COVID) study, we investigated cumulative fluid balance in invasively ventilated COVID-19 and ARDS patients and factors associated with a higher positive cumulative fluid balance. We aimed to test the association between the cumulative fluid balance during the first 4 calendar days of invasive ventilation and successful liberation of ventilation in these patients. We hypothesized that a higher cumulative fluid balance is independently associated with a lower probability of successful liberation of invasive ventilation in COVID-19 ARDS patients.

## Methods

### Design

PRoVENT-COVID is an investigator-initiated national, multicenter, observational cohort study that included COVID-19 patients with acute respiratory failure requiring invasive ventilation in 22 hospitals in the Netherlands in the first 3 months of the international outbreak. The study protocol was approved by the local institutional review board of Amsterdam University Medical Center (location ‘AMC’) and registered at Clinicaltrials.gov (NCT04346342). The institutional review board waived the requirement for written informed consent at the participating sites. The original study protocol was pre-published elsewhere [12]. The proposed plan and statistical analysis of the current analysis were approved by the Core Steering Committee and published with the website of PRoVENT-COVID before data acquisition [13]. The protocol was revised to address the unanticipated severely zero-inflated distribution of ventilator-free days during the initial data acquisition (Additional file 1: Fig. S1). This analysis adheres to the Strengthening the Reporting of Observational Studies in Epidemiology statement.

### Selection criteria

Invasively ventilated adult patients who met the criteria for ARDS using the Berlin definition [14] and who had real-time polymerase chain reaction confirmed SARS-CoV-2 infection admitted to one of the participating ICUs were eligible for participation. The original PRoVENT-COVID study protocol had no exclusion criteria; however, for the current analysis, we excluded patients if they were not invasively ventilated beyond the first 4 calendar days and patients who were transferred within the first 4 days of ventilation from or to another ICU that did not participate in the PRoVENT-COVID study.

### Exposure

The primary exposure of interest was the cumulative fluid balance. Cumulative fluid balance was obtained as a sum of daily fluid balance during the last 24-h, calculated by total fluid input minus total fluid output on a certain day of ICU admission for the first 4 calendar days of invasive ventilation. Insensible fluid loss such as perspiration or evaporative water loss due to respiration was not routinely measured and not included in the cumulative fluid balance calculation. Cumulative fluid balance from day 0 through day 1 was grouped as day 1, and the subsequent days were labelled as day 2 and day 3. Cumulative fluid balance during the first 4 calendar days is referred to hereafter as cumulative fluid balance at day 3.

### Outcomes

The primary outcome was successful liberation from invasive ventilation at 28 days. We chose this time frame following previous ARDS trials typically because either the subject died or extubated successfully by day 28 [15]. Secondary outcomes were acute kidney injury (according to a modified Kidney Disease Improving Global Outcomes definition) [16] and the need for renal replacement therapy after day 7. This variable was collected as dichotomous variable (yes/no) during follow-up at day 7, 28, and 90 [12]. Other secondary outcomes include duration of invasive ventilation in survivors and non-survivors, ICU and hospital length of stay in survivors and non-survivors, the incidence of tracheostomy in ICU, and 28-day mortality.

### Statistical analyses

Descriptive statistics were used to describe the study population and fluid management parameters. Data are presented as numbers and percentages for categorical variables and as means and standard deviation or median and interquartile range according to distribution. The normality of the distributions of quantitative variables was assessed by the Shapiro–Wilk test. Where appropriate, statistical variability is expressed by 95% confidence intervals.

Using a mixed effects model, we first examined the crude association between cumulative fluid balance and successful liberation from ventilation at day 28 with successful liberation of ventilation as a dependent variable, fluid balance as (fixed effect) as an independent variable, and hospital as a random intercept effect. To examine potential nonlinearity in the association, the cumulative fluid balance was entered as a restricted cubic spline function with 3 knots distributed equally along the density. The complexity of the spline function was reduced in a stepwise fashion until minimization of the Akaike information criterion (AIC) (Additional file 1: Fig. S2). The exposure (cumulative fluid balance at day 3) was divided into tertiles to facilitate interpretation.

The association between cumulative fluid balance at day 3 and the probability of successful liberation from invasive ventilation was then adjusted for possible confounders by including these variables as (fixed effect) covariates in the mixed effects model. Baseline physiological and laboratory variables (Day 0) were collected within one hour of ICU arrival or one hour of initiation of invasive ventilation, in accordance with the pre-published protocol [12]. The set of predefined adjustment variables included the following: sex, age, body mass index, serum creatinine, use of vasopressors (norepinephrine dose), tidal volume, arterial pH, positive end-expiratory pressure, partial pressure of oxygen to fraction inspired oxygen, dynamic respiratory system compliance and arterial lactate, all measured on the day of intubation.

Conditional on the assumption that the data were missing at random and the severity illness scores were collected differently by hospitals, before imputation, the percentage of missing data in the severity of illness scores in the first 3 days were assessed and addressed by a multi-level multiple imputation method. We imputed 20 datasets using multiple imputation by chained equations [17]. No exposure (day 3 fluid balance) or outcomes (survival and duration of ventilation) were imputed. All models described in the ‘statistical analysis’ section were reproduced in the 20 databases after multiple imputations, and the results were pooled. We considered statistical significance at *P* ≤ 0.05.

No formal statistical power calculation was conducted before the study. The sample size was solely based on the available data from the PRoVENT-COVID database.

### Sensitivity analyses

To assess the robustness of the findings toward the missing data and imputation method, we refit the main regression model (i.e., the marginal effect of day 3 fluid balance on the hazard of successful liberation from invasive ventilation) on cases with complete data only. To retain the largest possible sample size, only covariates that were significantly associated with the outcome in the main model were included in the sensitivity model.

We also estimated the main effect of different classes of ARDS severity (on the day of ICU admission) by including this variable in the adjusted mixed effects model.

## Results

### Patient population and characteristics

**Fig. 1:**
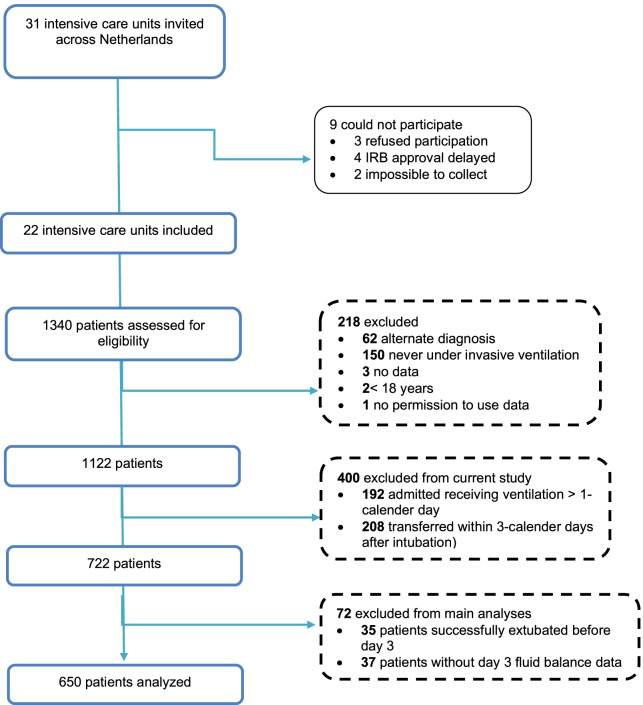
Flowchart of the study

We identified 687 invasively ventilated COVID-19 and ARDS patients admitted to ICUs between March 1, 2020, and June 1, 2020. The study flowchart is summarized in Fig. 1. Tables 1 and 2 describe the baseline, ventilation, and ICU characteristics of our study participants. The most prevalent comorbidities were hypertension and diabetes.

**Table 1:** Baseline characteristics of the included patient cohort

*N* (%)	Overall cohort	Lower	Intermediate	Higher	*P* value
	*N* = 650	215 (100.0)	220 (100.0)	215 (100.0)	
*Demographic characteristics*					
Gender, male, *N* (%)	467 (71.8)	145 (67.8)	160 (72.7)	161 (74.9)	0.243
Age, years	66.00 [58.00, 73.00]	65.00 [57.00, 71.00]	66.50 [58.75, 72.25]	66.00 [59.00, 73.00]	0.289
Weight, kg	86.00 [78.00, 96.00]	85.00 [75.00, 94.60]	86.00 [79.00, 96.00]	88.40 [80.00, 98.30]	0.023
Height, cm	175.00 [169.00, 182.00]	175.00 [168.00, 180.00]	175.00 [169.00, 182.00]	176.00 [170.00, 182.50]	0.341
Body mass index, kg/m^2^	27.78 [25.72, 30.86]	27.44 [25.06, 30.48]	27.99 [25.71, 30.47]	28.34 [26.02, 31.62]	0.081
*Comorbid conditions (%)*					
Comorbid. None	156 (24)	42 (19.6)	55 (25.0)	59 (27.4)	0.153
Hypertension	209 (32.2)	83 (38.8)	58 (26.4)	67 (31.2)	0.02
Heart failure	29 (4.5)	11 (5.1)	7 (3.2)	11 (5.1)	0.524
Diabetes mellitus	141 (21.7)	54 (25.2)	34 (15.5)	53 (24.7)	0.021
Chronic kidney disease	25 (3.8)	10 (4.7)	5 (2.3)	10 (4.7)	0.326
Liver cirrhosis	2 (0.3)	0 (0.0)	1 (0.5)	1 (0.5)	0.61
COPD	50 (7.7)	15 (7.0)	18 (8.2)	17 (7.9)	0.892
Hematological malignancy	10 (1.5)	3 (1.4)	4 (1.8)	3 (1.4)	0.919
Solid tumor malignancy	18 (2.8)	7 (3.3)	8 (3.6)	3 (1.4)	0.314
Neuromuscular disease	4 (0.6)	1 (0.5)	1 (0.5)	2 (0.9)	0.772
Immunosuppression use	10 (3.1)	7 (3.3)	6 (2.7)	7 (3.3)	0.932
Other comorbidities	315 (48.5)	108 (50.5)	108 (49.1)	99 (46.0)	0.644
Unknown comorbidities	2 (0.3)	1 (0.5)	1 (0.5)	0 (0.0)	0.608
Creatinine (µmol/L)	76.00 [61.00, 95.00]	74.00 [61.00, 93.00]	73.00 [57.50, 93.50]	79.00 [64.00, 103.25]	0.079

*COPD* chronic obstructive pulmonary disease

Data are shown as median [25th percentile, 75th percentile] or N (%). Non-normal values are displayed as median [25th percentile, 75th percentile], and those with normal distribution are represented as mean (standard deviation)

**Table 2:** Ventilator and other ICU variables on admission and follow-up days

*N* (%)	Overall cohort	Lower	Intermediate	Higher	*P* value
	*N* = 650	215 (100.0)	220 (100.0)	215 (100.0)	
*Day 0 variables (median [interquartile range])*					
Tidal volume (set)	410.50 [400.00, 480.00]	450.00 [400.00, 485.00]	400.00 [400.00, 420.00]	400.00 [380.00, 440.00]	0.271
Tidal volume (expired)	438.00 [387.00, 491.00]	434.50 [389.50, 476.25]	425.50 [379.25, 477.50]	450.00 [400.00, 508.00]	0.007
PEEP	12.00 [10.00, 15.00]	12.00 [10.00, 15.00]	12.00 [10.00, 14.00]	12.00 [10.00, 15.00]	0.125
Peak pressures	27.00 [24.00, 30.00]	28.00 [24.00, 31.00]	26.00 [24.00, 30.00]	27.00 [24.00, 30.00]	0.019
FiO_2_	0.70 [0.60, 0.85]	0.70 [0.60, 0.90]	0.65 [0.58, 0.80]	0.70 [0.60, 0.80]	0.105
pH	7.36 [7.30, 7.42]	7.36 [7.31, 7.41]	7.37 [7.31, 7.43]	7.36 [7.29, 7.41]	0.428
PaO_2_	10.88 [9.40, 13.00]	10.80 [9.50, 12.66]	10.80 [9.00, 13.15]	10.90 [9.50, 13.50]	0.682
PaO_2_/FiO_2_ ratio	123.00 [94.69, 165.66]	120.00 [96.59,153.92]	121.88 [92.78, 176.87]	130.03 [93.33, 171.25]	0.126
Dynamic compliance (Cdyn)	30.16 [24.16, 37.23]	28.62 [22.79, 34.62]	30.36 [23.79, 37.51]	31.38 [26.10, 40.77]	< 0.001
Lactate	1.20 [0.90, 1.50]	1.2 [0.97, 1.50]	1.20 [0.90, 1.50]	1.10 [0.90, 1.40]	0.084
SAPS	34.50 [28.75, 43.00]	32.00 [27.00, 38.00]	32.00 [26.50, 39.50]	38.00 [32.00, 45.50]	0.001
APACHE II	17.00 [12.00, 22.00]	16.00 [12.00, 22.00]	17.00 [11.50, 22.00]	16.00 [13.25, 19.75]	0.979
APACHE IV	56.95 [45.00, 69.00]	51.00 [43.00, 65.25]	56.50 [44.00, 65.00]	59.00 [46.00, 72.50]	0.083
SOFA	7.00 [6.00, 10.00]	7.00 [6.00, 10.00]	7.00 [6.00, 9.00]	8.00 [6.00, 9.00]	0.489
Creatinine (µmol/L)	73.00 [58.00, 93.00]	70.00 [57.00, 87.00]	73.00 [57.50, 93.50]	75.00 [63.00, 99.50]	0.055
Daily administered fluid (ml)	923.64 [298.50, 1744.25]	1131.50 [345.60, 1944.25]	784.05 [250.00, 1594.50]	875.00 [290.50, 1733.50]	0.034
Urine (ml)	780.00 [417.50, 1200.00]	780.00 [450.00, 1220.00]	825.00 [460.00, 1240.00]	725.00 [398.75, 1130.00]	0.152
Urine (ml/kg)	8.57 [4.71, 14.00]	9.07 [4.88, 14.67]	8.96 [5.11, 14.40]	7.76 [4.45, 12.69]	0.043
Ventilator-free days	3.00 [0.00, 11.00]	3.00 [0.00, 9.00]	4.00 [0.00, 11.25]	0.00 [0.00, 12.00]	0.286
Cumulative fluid balance day 0 (ml)	661.50 [91.33, 1496.00]	698.00 [− 34.00, 1598.00]	556.00 [37.50, 1283.10]	826.00 [212.00, 1762.00]	0.016
Number of patients on norepinephrine	649 (99.8)	213 (99.5)	220 (100)	215 (100)	0.361
Norepinephrine dose, mg	0.45 [0.01, 3.37]	0.04 [0.01, 2.17]	0.78 [0.01, 3.40]	0.89 [0.01, 4.14]	0.005
*Day 1 variables*					
SOFA	7.00 [6.00, 9.00]	7.00 [6.00, 9.00]	7.00 [5.25, 9.00]	7.00 [6.00, 9.00]	0.373
Creatinine (µmol/L)	84.00 [66.00, 118.00]	83.00 [66.00, 123.00]	80.00 [63.00, 111.00]	86.00 [68.00, 121.75]	0.171
Daily administered fluid (ml)	1670.50 [743.70, 2577.50]	1758.50 [848.05, 2588.50]	1435.50 [644.58, 2299.25]	1725.00 [750.00, 2851.35]	0.152
Urine (ml)	780.00 [417.50, 1200.00]	1125.00 [778.75, 1588.25]	1062.50 [805.00, 1446.25]	1202.02(697.48)	0.759
Urine (ml/kg)	8.57 [4.71, 14.00]	13.50 [8.92, 19.07]	12.62 [8.76, 17.38]	11.88 [8.40, 17.58]	0.043
Cumulative fluid balance (ml) day 1	1621.00 [871.42, 2382.25]	1460.90 [649.70, 21,621.00]	1507.50 [824.12, 2116.50]	1918.00 [1173.35, 2867.45]	< 0.001
Number of patients on norepinephrine	576 (88.6)	186 (86.9)	189 (85.9)	200 (93)	0.042
Norepinephrine, mg/24-h	11.20 [4.44, 49.96]	11.98 [3.60, 9216.00]	8.99 [3.46, 21.56]	12.21 [6.00, 41.40]	0.021
*Day 2 variables*					
SOFA	7.00 [6.00, 10.00]	7.00 [6.00, 11.00]	7.00 [5.00, 9.00]	7.00 [6.00, 9.00]	0.232
Creatinine (µmol/L)	85.00 [66.00, 133.00]	86.00 [64.00, 131.00]	83.00 [63.75, 124.25]	87.00 [70.00, 143.00]	0.215
Daily administered fluid (ml)	1243.15 [460.90, 1997.75]	1288.50 [476.28, 1936.75]	1169.00 [411.67, 1822.57]	1230.00 [511.00, 2252.40]	0.191
Urine (ml)	1075.00 [774.00, 1503.25]	1407.50 [990.00, 2071.25]	1095.00 [833.75, 1437.75]	1121.19 (696.37)	< 0.001
Urine (ml/kg)	12.56 [8.74, 17.75]	17.19 [11.00, 24.66]	12.96 [8.97, 17.48]	11.00 [8.39, 16.16]	< 0.001
Cumulative fluid balance (ml) day 2	1241.95 [548.75, 1979.75]	773.50 [0.25, 1398.75]	1157.00 [615.00, 1807.25]	1782.00 [1219.50, 2530.00]	< 0.001
Number of patients on norepinephrine	551 (84.8)	167 (78)	187 (85)	196 (91.2)	0.001
Norepinephrine, mg/24-h	10.70 [4.12, 86.25]	12.40 [3.74, 9216.00]	7.92 [3.33, 26.64]	14.65 [5.11, 41.00]	0.017
*Day 3 variables*					
SOFA	7.00 [6.00, 10.00]	7.00 [6.00, 11.00]	7.00 [5.00, 9.00]	7.00 [6.00, 9.00]	0.56
Creatinine (µmol/L)	84.00 [63.00, 140.00]	85.00 [62.00, 129.00]	79.50 [61.00, 127.50]	86.00 [68.00, 171.00]	0.07
Daily administered fluid (ml)	956.40 [276.75, 1719.25]	977.98 [252.50, 1555.75]	650.70 [238.00, 1607.60]	1189.90 [504.00, 2160.00]	< 0.001
Urine (ml)	1155.00 [830.00, 1640.00]	2112.50 [1511.25, 2735.00]	1280.00 [867.50, 1722.50]	1030.17 (641.13)	< 0.001
Urine (ml/kg)	13.33 [9.23, 19.22]	24.04 [17.82, 32.83]	15.05 [10.43, 20.24]	11.23 [7.93, 14.72]	< 0.001
Cumulative fluid balance (ml) day 3	780.00 [7.47, 1623.25]	− 344.75 [− 751.55, 1.72]	780.00 [499.72, 1013.75]	1988.00 [1638.30, 2397.00]	< 0.001
Number of patients on norepinephrine	499 (76.8)	144 (67.3)	168 (76.4)	186 (86.5)	< 0.001
Norepinephrine, mg/24-h	9.90 [3.30, 91.78]	8.75 [2.70, 9216.00]	6.90 [2.45, 19.74]	13.75 [5.18, 58.13]	0.003

*APACHE* Acute Physiology Assessment and Chronic Health Evaluation, *FiO2* fraction of inspired oxygen, *PaO2* arterial partial pressure of oxygen, *PEEP* positive end-expiratory pressure, *SAPS* Simplified Acute Physiology Score, *SOFA* Sequential Organ Failure Assessment

Data are shown as median [25th percentile, 75th percentile] or N (%). Non-normal values are displayed as median [25th percentile, 75th percentile], and those with normal distribution are represented as mean (standard deviation)

### Cumulative fluid balance distribution

The distribution of cumulative fluid balance at day 3 was evaluated for the overall cohort (Additional file 1: Fig. S3). Complete exposure (fluid balance) data were available for 676 subjects on day 0; 673 (99.5%) subjects on day 1; 661 (97.7%) subjects on day 2; and 650 (96.1%) subjects on day 3. Patients were divided into tertiles by day 3 cumulative fluid balance: highest, intermediate, and lowest tertile groups had a median cumulative fluid balance of 1.98 L [range 1.27–7.72 L], 0.78 L [0.26–1.27 L], and − 0.35 L [− 6.52–0.26 L], respectively. Patients in the lower cumulative fluid balance group had a higher prevalence of chronic hypertension and diabetes mellitus, whereas patients in the higher cumulative fluid balance group were noted to have worse baseline Simplified Acute Physiology Score (Table 1).

### Association of cumulative fluid balance with outcomes

The association between day 3 cumulative fluid balance and the hazard of successful liberation from ventilation was most parsimoniously characterized by a 0-spline (linear) survival model. Models with 3-, 2-, or 1-knot-restricted cubic splines for cumulative fluid balance had higher AICs (i.e., no better fit) (Additional file 1: Fig. S2).

**Fig. 2:**
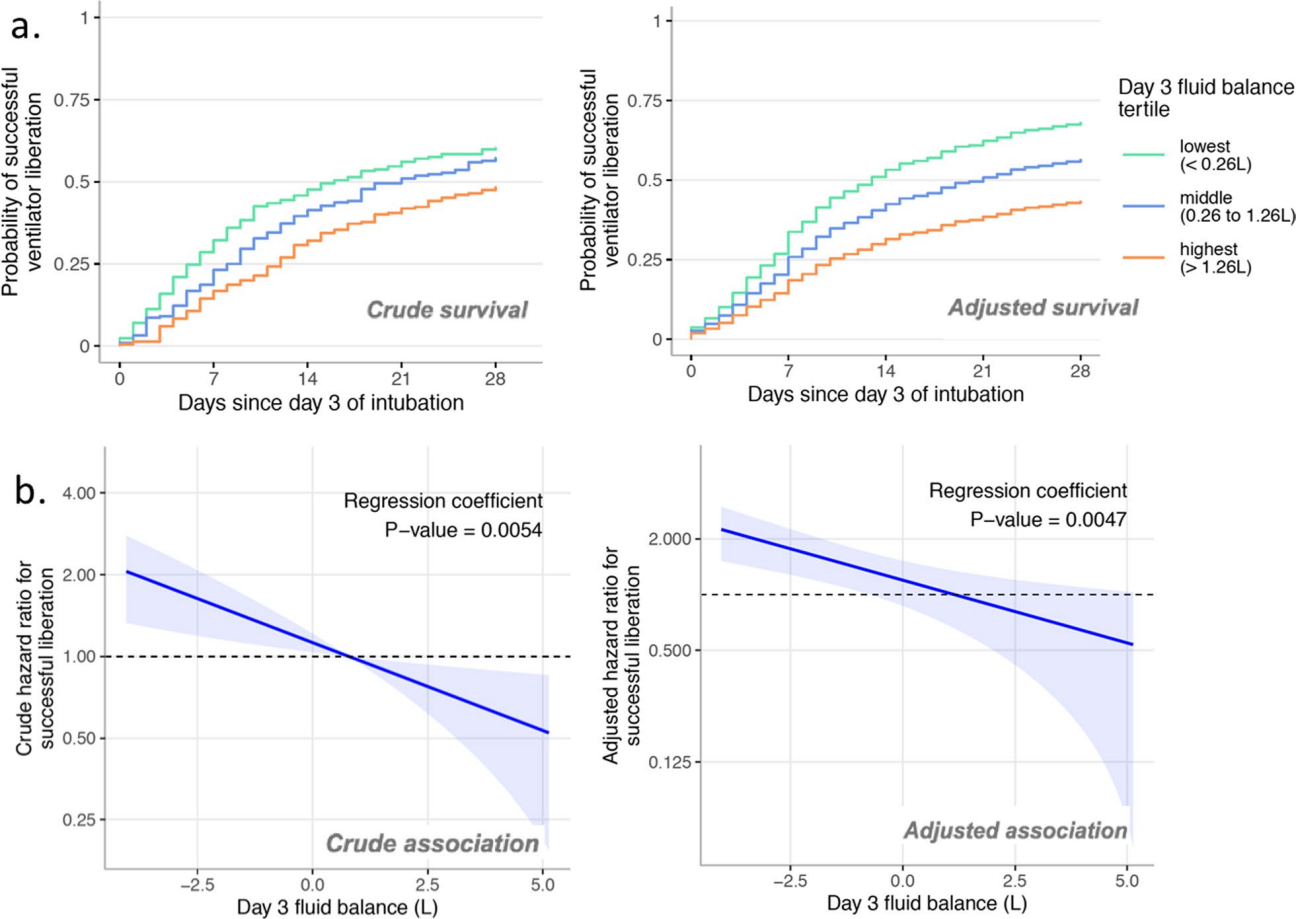
Survival and marginal effect plots. **a** Survival plots (unadjusted and adjusted) showing predicted probability of successful liberation of invasive ventilation as a function of day 3 fluid balance—separated in tertiles. **b** Marginal effect (unadjusted and adjusted) of day 3 cumulative fluid balance on the hazard of successful liberation from invasive ventilation after adjustment for predefined confounding variables. A higher day 3 cumulative fluid balance was associated with a lower hazard (i.e., a lower probability over time) of successful liberation

The resulting association between cumulative fluid balance at day 3 and the probability of successful liberation from invasive ventilation is shown in Fig. 2. In unadjusted analysis, there was a significant association between higher cumulative fluid balance at day 3 and a lower probability of successful liberation from ventilation, with a hazard ratio per liter fluid balance of 0.86 (95% CI 0.78–0.96, *P* = 0.005). After adjusting for a predefined set of possible confounding variables (listed in Additional file 1: Table S1a), exposure to higher cumulative fluid balance at day 3 remained significantly associated with lower probability of successful liberation of invasive ventilation. The adjusted hazard ratio for successful liberation from invasive ventilation associated with each liter increase in cumulative fluid balance was 0.86 (95% CI 0.77–0.95, *P* = 0.0047). In the post hoc analysis, we assessed the impact of imbalances of chronic baseline hypertension and diabetes mellitus between the tertiles groups and found no difference in the main effect estimate HR 0.83 (95%CI 0.73;0.94) compared to 0.86 (95% CI 0.78;0.96) for the model without hypertension and diabetes (listed in Additional file 1: Table S1b).

### Secondary outcomes and sensitivity analysis

The secondary outcomes are summarized in Table 3. Length of ICU stay, length of hospital stays, and duration of intubation were significantly shorter for surviving patients who were in the lower tertile of cumulative fluid balance. Other outcomes did not differ significantly between fluid balance tertiles.

**Table 3:** Patient-centered endpoints stratified by tertiles

	Lower	Intermediate	Higher	*P* value
Overall cohort, *N* = 650, *N* (%)	215 (100.0)	220 (100.0)	215 (100.0)	
Acute kidney injury	110 (52.1)	102 (46.6)	122 (56.7)	0.105
Requiring RRT	63 (29.4)	58 (26.4)	67 (31.2)	0.535
CVVH/CVVHDF	46 (21.5)	43 (19.5)	55 (25.6)	0.304
Hemodialysis	3 (1.4)	7 (3.2)	7 (3.3)	0.395
Peritoneal dialysis	0 (0.0)	0(0.0)	1 (0.5)	0.364
28-day mortality	62 (29.0)	61 (27.7)	78 (36.3)	0.116
Duration of intubation at 28 days	8.00 [4.00, 16.00]	11.00 [5.00, 21.00]	11.00 [4.00, 21.00]	**0.019**
Survivors	8.00 [4.00, 18.25]	12.00 [6.00, 26.00]	14.00 [7.00, 28.00]	** < 0.001**
Non-survivors	6.50 [3.00, 13.75]	10.00 [2.00, 14.00]	5.50 [2.00, 11.00]	0.228
Tracheostomy incidence in ICU	36 (16.9%)	31 (14.1%)	43 (20%)	0.260
Intensive care unit length of stay	14.00 [9.00, 22.75]	18.00 [11.00, 28.00]	17.00 [9.50, 29.00]	0.019
Survivors	15.00 [9.75, 27.25]	20.00 [13.00, 34.00]	23.00 [14.00, 37.00]	** < 0.001**
Non-survivors	10.50 [7.00, 17.75]	14.00 [6.00, 18.00]	9.50 [6.00, 15.00]	0.249
Hospital length of stay	20.00 [13.00, 32.00]	24.00 [15.00, 38.00]	22.00 [12.00, 39.00]	0.076
Survivors	25.00 [16.00, 38.00]	30.00 [20.00, 44.00]	34.00 [21.50, 47.50]	**0.001**
Non-survivors	10.50 [7.00, 17.75]	15.00 [6.00, 18.00]	9.50 [6.00, 15.00]	0.192

*CVVHD* Continuous veno-venous hemodiafiltration, *CVVH* continuous veno-venous hemofiltration, *RRT* renal replacement therapy

Data are shown as median [25th percentile, 75th percentile] or N (%). *P* values in bold text indicate statistical significance at *P* < 0.05

Two sensitivity analyses were performed. First, the robustness of our findings was assessed toward the missing data and imputation method (Additional file 1: Table S2). In the complete-cases-only model, the sample size was reduced to 461 patients; the estimated association between day 3 cumulative fluid balance and successful liberation of invasive ventilation was similar to the estimation with imputed data. The hazard ratio per liter fluid balance was consistent with the primary analysis, 0.86 (95% CI 0.76–0.98, *P* = 0.0247).

**Fig. 3:**
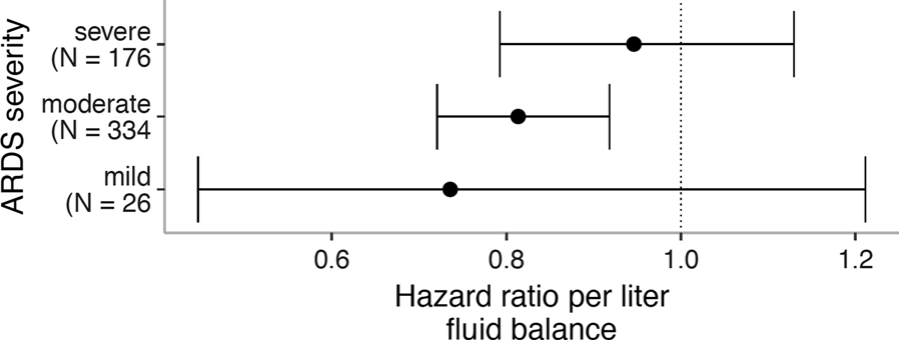
Sensitivity analysis showing the consistency of main effect over ARDS severity. *ARDS* acute respiratory distress syndrome

Second, the models were re-analyzed according to ARDS severity on the ICU admission day, considering a possible interaction between the severity of ARDS and fluid balance. The interaction between ARDS severity and day 3 cumulative fluid balance did not improve the model fit (AIC of interaction model 4336 vs. 4335 for reduced model), indicating that there was no significant interaction between day 3 cumulative fluid balance and ARDS severity on the association with successful liberation from invasive ventilation (Fig. 3).

## Discussion

The main findings of this multicentric observational study of COVID-19 and ARDS patients include the following: (1) A higher day 3 cumulative fluid balance was associated with a lower probability of successful liberation from invasive ventilation by day 28; (2) these results remained consistent even after adjustment for potential predefined confounding factors and sensitivity analyses; and (3) reduction in duration of invasive ventilation and hospital and ICU length of stay was noted in patients who had lower cumulative fluid balance.

Our results add to the growing evidence suggesting the unfavorable effect of higher positive fluid balance on outcomes in critically ill patients [18–21]. However, compared to these studies, there are also some notable differences in our study. We specifically evaluated the exposure of cumulative fluid balance in COVID-19 and ARDS patients on the ventilation liberation irrespective of prior spontaneous breathing trials. Cumulative fluid balance was calculated from hospital admission until day 3, whereas in other studies, it was calculated differently. Despite these differences, a signal of potential harm with excessive cumulative fluid balance and weaning outcomes was consistently observed.

Evidence emanating from large trials of ARDS patients has led to an overall practice change that resulted in relatively less aggressive initial fluid management. In 2006, the Fluids and Catheters Treatment Trial reported a causal effect between positive fluid balance and duration of ventilation in ARDS patients [5]. The authors found that the conservative group had a shorter ventilation duration than the liberal-strategy group without an increase in non-pulmonary organ failure. Another study, performed by the ARDSnet, showed that negative cumulative fluid balance was significantly associated with more ventilator-free days and lower mortality than positive cumulative fluid balance [22]. A limitation of using ‘ventilator-free days’ in these reports is that a more frequently occurring component of the composite (such as survival or duration of ventilation) presumably drives the effect estimates and could influence the results, even stronger when the components are oppositely affected by the exposure [1, 15]. Our rationale for using ‘ventilator-free days’ was to compare our analysis to previously conducted studies readily; one of the challenges was disentangling the contribution of ‘zero-inflated distribution’ in ventilator-free days. However, it is possible that a greater-than-expected number of non-survivors had died within 24-h of initiation of ventilation, and this could presumably drive the mean difference toward null. Or, because of unknown factors, certain patients might not have been able to present values other than zero. Nevertheless, we addressed it by restricting our primary outcome to only ‘successful ventilation liberation’ instead of ‘ventilator-free days’; however, our analysis suffered from model selection bias.

Several mechanisms may explain the association of higher early cumulative fluid balance and decreased odds of ventilation liberation. Higher positive fluid balance increases the extravascular lung water, and inattention to fluid overload may inadvertently promote counterproductive outcomes, such as pulmonary vascular dysregulation and alveolar edema, contributing to weaning failure. This risk is particularly high among patients with COVID-19 and ARDS because of relatively higher extravascular lung water and pulmonary vascular permeability indices, in distinct contrast to non-COVID ARDS [23]. Furthermore, alveolar fluid clearance is perhaps slow or even impaired in ARDS pathophysiology. The combined processes of high vascular permeability and impaired alveolar fluid clearance may therefore rapidly worsen the alveolar edema—even with a slight increase in intravascular volume [24, 25]. Consistent with this view, we showed that even a one-liter increase in the dose of cumulative fluid balance might significantly decrease ventilation liberation odds. For example, about 14% (hazard ratio of 0.86) lower rate of successful ventilation liberation was noted with each liter of fluid addition to cumulative fluid balance—implying a dose–response relationship. Importantly, our results do not imply a causal relationship, as causality can only be identified in a randomized trial; however, given the strength of association between cumulative fluid balance and weaning outcome, a well-designed trial seems well justified. Taken together with the previous research, our results indicate a possible beneficial effect of restrictive fluid management in invasively ventilated COVID-19 and ARDS patients.

Higher cumulative fluid balance has also been suggested in previous studies of non-COVID-19 ARDS patients to be potentially associated with worsened outcomes, such as acute kidney injury and decreased survival [26]. In our study, no association was observed in the lower cumulative fluid balance group with respect to our secondary endpoints, such as acute kidney injury, the requirement of renal replacement therapy, and mortality, with the caveat that our analysis was too small to evaluate these endpoints and, therefore, should be considered as only hypothesis-generating for future investigations.

The strengths of our study include the size of the multicenter cohort of 22 hospitals that comprised both academic and non-academic institutions, increasing the generalizability of our results. We took careful steps to prevent selection bias that could have been caused by patients who were transferred from other hospitals. Also, trained study coordinators performed careful data collection, and a pre-specified statistical analysis plan was prepared before data acquisition.

Our study is subject to several limitations. Although we adjusted for a limited set of pre-specified confounders, our results may be biased by a (large) number of unmeasured confounders. We considered variables observed after day 0 (such as cumulative vasopressor dose or ARDS severity on day 4) as potential mediators as they would presumably be influenced by the cumulative fluid balance and may be associated with the outcome. Adjusting for these variables would introduce bias through over adjustment [27, 28]. Our analysis did not account for race or ethnicity; the possible confounding effects on our primary outcome cannot be determined, therefore limiting our results’ generalizability and hindering our ability to examine racial disparity [29]. Significant heterogeneity exists in the resuscitation paradigm regarding the optimal time of initiation of diuretics in hemodynamically unstable patients. We acknowledge that the diuretics use, particularly in hemodynamically unstable patients requiring vasopressors, could be a confounder, not accounted for in our analysis.

Based on a priori-defined selection criteria, a trivial fraction (approximately 5%) of patients extubated at day 3 were excluded from the analysis (Fig. 1). It, therefore, remains unknown whether our findings apply to patients successfully liberated from invasive ventilation early in the course of the illness. To align with our pragmatic intent of evaluating the effectiveness of interventions in ‘usual care’ and their operational practicality in the initial months of the COVID-19 pandemic, dynamic parameters such as cardiac output/index and other advanced hemodynamic indices were not part of the a priori-defined collected variables. Multiple challenges to research existed during the pandemic that may have affected the clinical outcomes for the included patients, such as organizational issues to utilize resources to prevent future upheavals. Included data were derived from 22 collaborative hospitals that exhibited variation in practice; for example, weaning did not occur with a mandatory protocol, and healthcare provider-related bias could have affected the weaning outcomes. While the percentage of missing values was low, missing data-related bias due to the adoption of different severity illness scores by various centers was thoroughly handled by imputation approaches such as last observation carried forward and robust evaluation tools.

## Conclusions

This multicenter study of invasively ventilated COVID-19 and ARDS patients suggests a strong association between higher day 3 cumulative fluid balance and the duration of ventilation, even after adjusting for a predefined set of possible confounding variables. Nevertheless, randomized clinical trials are required to confirm our findings. To the extent that higher positive fluid balance suggests harm and influences weaning outcomes, maintenance of restrictive cumulative fluid balance may improve weaning outcomes in invasively ventilated COVID-19 and ARDS patients.

## Supplementary Information

**Additional file 1. Figure S1 a.** Histogram of model residuals showing zero-inflated count distributions. **b**: Predicted values vs. model residuals. **Figure S2.** Models with different spline complexities. **Figure S3.** Box and violin plots of cumulative fluid balance over days 0–3. **Table S1a.** Potential predefined confounding variables. **b**: Post-hoc analysis adjusting for diabetes and hypertension as possible confounding variables. **Table S2.** Sensitivity analysis to missing data and imputation method.

## Data Availability

The datasets used and/or analyzed during the current study are available from the authors on reasonable request.

## Abbreviations

AIC: Akaike information criterion
ARDS: Acute respiratory distress syndrome
COVID-19: Coronavirus disease 2019
ICU: Intensive care unit
PRoVENT-COVID: Practice of ventilation in COVID-19 patients
SARS-CoV-2: Severe acute respiratory syndrome coronavirus 2
