# Supplementary material for: Association between early cumulative fluid balance and successful liberation from invasive ventilation in COVID-19 ARDS patients — insights from the PRoVENT-COVID study: a national, multicenter, observational cohort analysis
Source: Crit Care. 2022 Jun 1;26:157. doi: 10.1186/s13054-022-04023-y (PMC9157033; doi:10.1186/s13054-022-04023-y)
Supplement: Supplementary file 1 — Additional file 1. Figure S1 a. Histogram of model residuals showing zero-inflated count distributions. b: Predicted values vs. model residuals. Figure S2. Models with different spline complexities. Figure S3. Box and violin plots of cumulative fluid balance over days 0–3. Table S1a. Potential predefined confounding variables. b: Post-hoc analysis adjusting for diabetes and hypertension as possible confounding variables. Table S2. Sensitivity analysis to missing data and imputation method. [file 13054_2022_4023_MOESM1_ESM.docx]

**Association between Early Cumulative Fluid Balance and Successful Liberation from Invasive Ventilation in COVID–19 ARDS Patients – insights from the PRoVENT–COVID study: a national, multicenter, observational cohort analysis**

**Additional File 1**

Sanchit Ahuja^1,2^, Harm-Jan de Grooth^3^, Frederique Paulus^4,5^, Fleur L. van der Ven^4^, Ary Serpa Neto^6,7,8^, Marcus J. Schultz^4,9,10^, Pieter R. Tuinman^3^, for the PRoVENT-COVID Study Collaborative Group^*^ ‘PRactice of VENTilation in COVID–19’

^1^Department of Anesthesiology, Pain Management & Perioperative Medicine, Henry Ford Hospital, Detroit, Michigan, USA

^2^Outcomes Research Consortium, Cleveland Clinic, Cleveland, Ohio, USA

^3^Department of Intensive Care, Amsterdam UMC, location VU Medical Center, Amsterdam, The Netherlands

^4^Department of Intensive Care, Amsterdam UMC, location AMC, Amsterdam, The Netherlands

^5^ACHIEVE, Centre of Applied Research, Amsterdam University of Applied Sciences, Faculty of Health, Amsterdam, The Netherlands

^6^Department of Critical Care Medicine, Melbourne Medical School, University of Melbourne, Austin Hospital, Melbourne, Australia.

^7^Australian and New Zealand Intensive Care Research Centre (ANZIC-RC), School of Public Health and Preventive Medicine, Monash University, Melbourne, Australia.

^8^Department of Critical Care Medicine, Hospital Israelita Albert Einstein, São Paulo, Brazil.

^9^Mahidol Oxford Tropical Medicine Research Unit (MORU), Mahidol University, Bangkok, Thailand

^10^Nuffield Department of Medicine, University of Oxford, Oxford, UK

**Correspondence:**

Prof. Marcus J. Schultz, MD PhD

Department of Intensive Care, C3–415, Academic Medical Center, Meibergdreef 9

1105 AZ Amsterdam, The Netherlands

Email: [m.j.schultz@amsterdamumc.nl](mailto:m.j.schultz@amsterdamumc.nl)

**Table of Contents:**

**Supplementary Figure 1 a:** Histogram of model residuals showing zero-inflated count

distributions. **b:** Predicted values vs. model residuals.

**Supplementary Figure 2:** Models with different spline complexities

**Supplementary Figure 3:** Box and violin plots of cumulative fluid balance over days 0-3

**Supplementary Table 1a:** Potential predefined confounding variables**. b:** Post-hoc analysis adjusting for diabetes and hypertension as possible confounding variables

**Supplementary Table 2** Sensitivity analysis to missing data and imputation method


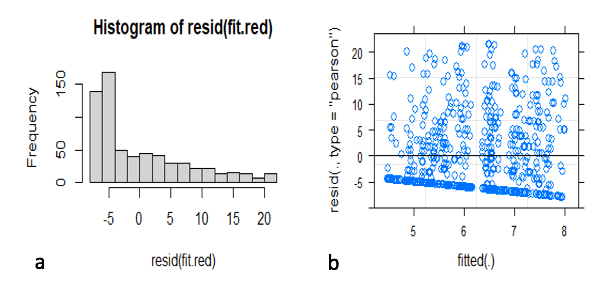


**Supplementary Figure 1**: After fitting the prespecified linear model regressing ventilator-free days on cumulative fluid balance at day 3, the residuals were found to be poorly distributed because of the relative overabundance of cases with 0 ventilator-free days (i.e., the distribution was severely zero-inflated). Based on these findings, this model was abandoned and all reported results were based on other methods. **a** Histogram of model residuals showing severe skewness as a consequence of zero-inflated count distributions. **b** Predicted values vs. model residuals.


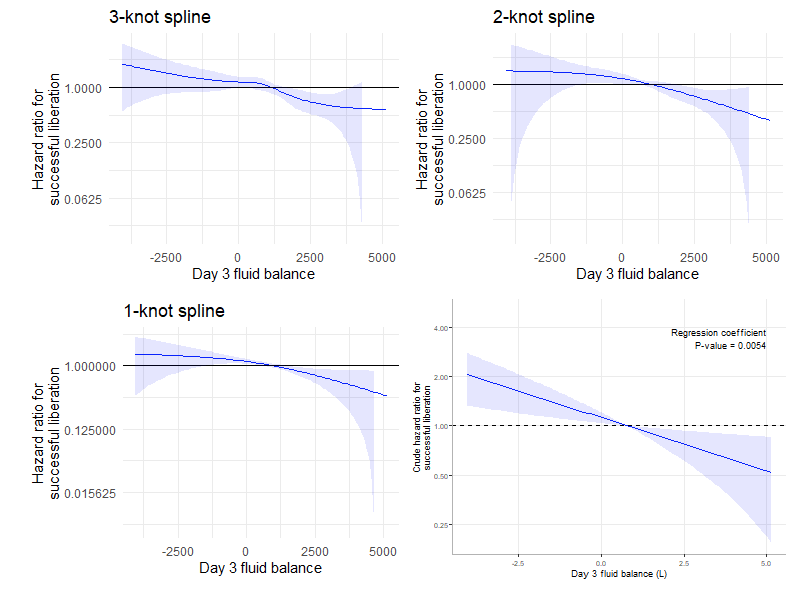


**Supplementary Figure 2:** Models regressing the hazard of successful liberation on day 3 fluid balance with different spline complexities. The Akaike Information Criterion (AIC) for the four models was 4381, 4379, 4377 and 4376, in descending order of model complexity. Therefore, the no-spline model (AIC 4376) was found to be most parsimonious and was used for the main analyses.


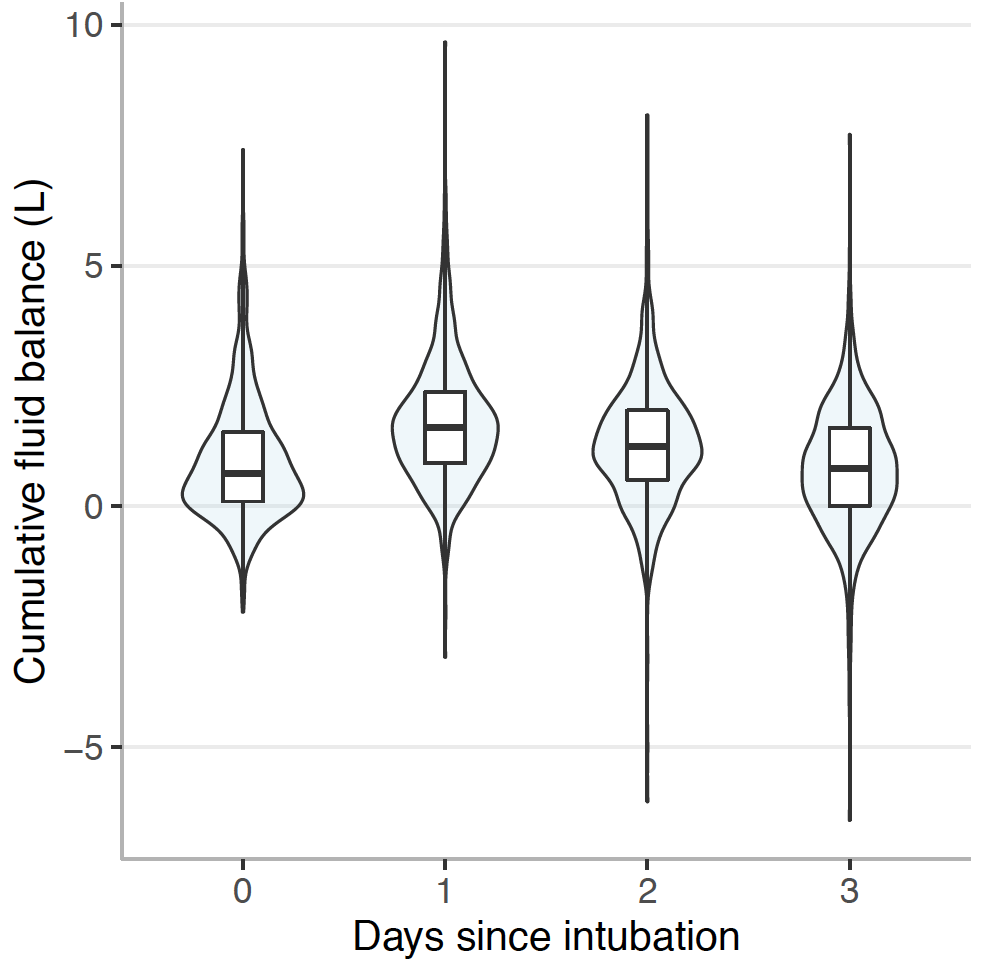


**Supplementary Figure 3**: Box and violin plots of cumulative fluid balance over days 0-3

**Supplementary Table 1a**: Potential prespecified confounding variables

| **Confounding Variables** | **Hazards ratio, (95% CI)** | **P value** |
| --- | --- | --- |
| cFB at day 3, ml | 0.99, (0.999-0.999) | 0.002 |
| Sex | 0.78, (0.580-1.070) | 0.127 |
| Age | 0.95, (0.946-0.966) | 0.001 |
| Body mass index (kg/m^2^) | 0.99, (0.977-1.015) | 0.725 |
| Creatinine at day 0 | 0.99, (0.990-0.998) | 0.012 |
| Norepinephrine dose, calculated log dose at day 0 | 1.02, (0.979-1.077) | 0.267 |
| Tidal volume at day 0 (expired) | 1.00, (0.998-1.001) | 0.994 |
| pH at day 0 | 13.5, (4.721-38.901) | 0.001 |
| Heart rate at day 0 | 0.99, (0.991-1.002) | 0.294 |
| PEEP at day 0 | 0.97, (0.927-1.017) | 0.223 |
| PaO_2_/FiO_2_ at day 0 | 0.99, (0.996-0.999) | 0.034 |
| Dynamic compliance (Cdyn) at day 0 | 1.00, (0.996-1.014) | 0.209 |
| Lactate at day 0 | 1.00, (0.881-1.153) | 0.902 |

The hazards ratios were estimated using Cox proportional hazard model, adjusted for predefined confounding variables. Abbreviations. cFB cumulative fluid balance, PEEP positive endexpiratory pressure, PaO2/FiO2 PaO2 arterial partial pressure of oxygen/Fraction of inspired oxygen. Note that the hazard ratio for day 3 cFB is expressed here per milliliter.

**Supplementary Table 1b: Post-hoc analysis adjusting for diabetes and hypertension as possible confounding variables**

| **Confounding Variables** | **Hazards ratio, (95% CI)** | **P value** |
| --- | --- | --- |
| Diabetes mellitus | 0.948, (0.730-1.230) | 0.688 |
| Hypertension | 0.919, (0.776-1.089) | 0.333 |

**Supplementary Table 2:** Senstivity analysis to missing data and imputation method

|  | **Hazards ratio, (95% CI)** | **P value** |
| --- | --- | --- |
| cFB at day 3, ml | 0.99, (0.99, 1.00) | 0.02 |
| Sex | 0.819, (0.60, 1.11) | 0.2 |
| Age | 0.956, (0.94, 0.96) | <0.001 |
| Creatinine at day 0 | 0.994, (0.989, 0.987) | 1 |
| pH at day 0 | 18.1444, (3.58, 91.74) | 0.0004 |
| PaO_2_/FiO_2_ | 0.9987, (0.99, 1.00) | 0.15 |

The hazards ratios were estimated using Cox proportional hazard model, adjusted for missing data and imputation method. Abbreviations. cFB cumulative fluid balance, PaO2/FiO2 PaO2 arterial partial pressure of oxygen/Fraction of inspired oxygen. Note that the hazard ratio for day 3 cFB is expressed here per milliliter.

*Acknowledgements*: ***PRoVENT-COVID Collaborative Group.** *Investigators:* (in alphabetic order): S. Ahuja^1^; J.P. van Akkeren^2^; A.G. Algera^3^; C.K. Algoe^3^; R.B. van Amstel^3^; A. Artigas^4^; O.L. Baur^3^; P. van de Berg^5^; A.E. van den Berg^6^; D.C.J.J. Bergmans^7^; D.I. van den Bersselaar^3^; F.A. Bertens^3^; A.J.G.H. Bindels^5^; M.M. de Boer^3^; S. den Boer^8^; L.S. Boers^3^; M. Bogerd^3^; L.D.J. Bos^3^; M. Botta^3^; J.S. Breel^9^; H. de Bruin^3^; S. de Bruin^3^; C.L. Bruna^3^; L.A. Buiteman–Kruizinga^10^; O. L. Cremer^11^; R.M. Determann^12^; W. Dieperink^13^; D.A. Dongelmans^3^; H.S. Franke^13^; M.S. Galek-Aldridge^3^; M.J. de Graaff^14^; L.A. Hagens^3^; J.J. Haringman^16^; S.T. van der Heide^3^; P.L.J. van der Heiden^10^; N.F.L. Heijnen^15^; S.J.P. Hiel^2^; L.L. Hoeijmakers^3^; L. Hol^3,9^; M.W. Hollmann^9^; M.E. Hoogendoorn^16^; J. Horn^3^; R. van der Horst^17^; E.L.K. Ie^3^; D. Ivanov^3^; N.P. Juffermans^12^; E. Kho^3^; E.S. de Klerk^9^; A.W.M.M. Koopman-van Gemert^18^; M. Koopmans^12^; S. Kucukcelebi^3^; M.A. Kuiper^19^; D.W. de Lange^11^; N. van Mourik^3^; S.G. Nijbroek^3,9^; M. Onrust^13^; E.A.N. Oostdijk^20^; F. Paulus^3,21^; C.J. Pennartz^3^; J. Pillay^3,13^; L. Pisani^3^; I.M. Purmer^6^; T.C.D. Rettig^22^; J.P. Roozeman^3^; M.T.U. Schuijt^3^; M.J. Schultz^3,23,24^; A. Serpa Neto^25^; M.E. Sleeswijk^26^; M.R. Smit^3^; P.E. Spronk^27^; W. Stilma^3^; A.C. Strang^28^; A.M. Tsonas^3^; P.R. Tuinman^29^; C.M.A. Valk^3^; F.L. Veen-Schra^16^; L.I. Veldhuis^3^; P. van Velzen^30^; W.H. van der Ven^9^; A.P.J. Vlaar^3^; P. van Vliet^31^; P.H.J. van der Voort^13^; L. van Welie^32^; H.J.F.T. Wesselink^16^; H.H. van der Wier-Lubbers^16^; B. van Wijk^3^; T. Winters^3^; W.Y. Wong^3^; A.R.H. van Zanten^32^.

*Institutional and Departmental Affiliations:* ^1^Department of Anesthesiology, Pain Management & Perioperative Medicine**, Henry Ford Health System, Detroit, Michigan, United States;** ^2^Department of Intensive Care**, Maxima Medical Center, Eindhoven, The Netherlands**; ^3^Department of Intensive Care, **Amsterdam University Medical Centers, location ‘Academic Medical Center’, Amsterdam, The Netherlands**; ^4^Critical Care Center, **Sabadell Hospital, Sabadell, Spain**; ^5^Department of Intensive Care, **Catharina Hospital**, **Eindhoven, The Netherlands**; ^6^Department of Intensive Care, **Haga Hospital, the Hague, The Netherlands;** ^7^Department of Intensive Care, **Maastricht University Medical Center, Maastricht, The Netherlands**; ^8^Department of Intensive Care, **Spaarne Hospital, Haarlem, The Netherlands**; ^9^Department of Anaesthesiology, **Amsterdam University Medical Centers, location ‘Academic Medical Center’, Amsterdam, The Netherlands**; ^10^Department of Intensive Care, **Reinier de Graaf Hospital, Delft, The Netherlands**; ^11^Department of Intensive Care, **University Medical Center Utrecht, Utrecht, The Netherlands**; ^12^Department of Intensive Care, **OLVG Hospital, location East, Amsterdam, The Netherlands**; ^13^Department of Intensive Care, **University Medical Center Groningen, Groningen, The Netherlands**; ^14^Department of Intensive Care, **Sint Antonius Hospital, Nieuwegein, The Netherlands**; ^15^Department of Intensive Care, Maastricht University Medical Center, **Maastricht, The Netherlands;** ^16^ Department of Intensive Care, **Isala Hospital, Zwolle, The Netherlands**; ^17^Department of Intensive Care; **Zuyderland Hospital, Heerlen and Sittard, The Netherlands**; ^18^Department of Intensive Care **ZGT Hospital, Almelo, The Netherlands**; ^19^Department of Intensive Care **Medical Center Leeuwarden, Leeuwarden, The Netherlands**; ^20^Department of Intensive Care; **Maasstad Hospital, Rotterdam, The Netherlands**; ^21^ACHIEVE, Center of Applied Research **Amsterdam University of Applied Sciences, Faculty of Health, Amsterdam, The Netherlands**; ^22^Department of Intensive Care **Amphia Hospital, Breda, The Netherlands**; ^23^Mahidol–Oxford Tropical Medicine Research Unit (MORU), **Mahidol University, Bangkok, Thailand**; ^24^Nuffield Department of Medicine, **University of Oxford, Oxford, United Kingdom**; ^25^Department of Critical Care Medicine, **Australian and New Zealand Intensive Care Research Center (ANZIC-RC), Monash University, Melbourne, Australia**; ^26^Department of Intensive Care, **FlevoHospital, Almere, The Netherlands**; ^27^Department of Intensive Care, **Gelre Hospital, Apeldoorn and Zutphen, The Netherlands**:; ^28^Department of Intensive Care, **Rijnstate Hospital, Arnhem, The Netherlands**; ^29^Department of Intensive Care, **Amsterdam University Medical Centers, location ‘VU Medical Center’, Amsterdam, The Netherlands**; ^30^Department of Intensive Care, **Dijklander Hospital, location Hoorn, Hoorn, The Netherlands**; ^31^Department of Intensive Care **Haaglanden Medical Center, location Westeinde, the Hague, The Netherlands**; ^32^Department of Intensive Care **Gelderse Vallei Hospital, Ede, The Netherlands**.
